# Severe denosumab-induced hypocalcemia requiring long-term intensified medication in a patient with *EGFR*-mutant lung cancer and diffuse osteoblastic bone metastases

**DOI:** 10.1016/j.rmcr.2025.102183

**Published:** 2025-02-26

**Authors:** Masayuki Mori, Masayuki Shirasawa, Akihito Oguri, Hiroki Yamamoto, Hideaki Manabe, Yoshiro Nakahara, Takashi Sato, Katsuhiko Naoki

**Affiliations:** a Department of Respiratory Medicine, School of Medicine, Kitasato University, Kanagawa, Japan. Address: 1-15-1 Kitasato, Minami, Sagamihara, Kanagawa, 252-0375, Japan; b Department of Respiratory Medicine, Tachikawa Sogo Hospital, Tokyo, Japan. Address: 4-1, Midori, Tachikawa, Tokyo, 190-8578, Japan; c Department of Respiratory Medicine, Sagamihara Kyodo Hospital, Kanagawa, Japan. Address: 4-3-1 Hashimotodai, Midori, Sagamihara, Kanagawa, 252-5188, Japan

**Keywords:** Denosumab, Hypocalcemia, Lung adenocarcinoma, Bone metastasis, Tartrate-resistant acid phosphatase-5b (TRACP-5b)

## Abstract

Lung cancer often causes bone metastasis, and denosumab is administered to bone metastases to prevent bone-related adverse events. One of the important side effects of denosumab is hypocalcemia, but this is generally not a problem, as it is used with calcium supplementation. A 48-year-old non-smoker male was diagnosed with lung adenocarcinoma with EGFR L858R mutation with diffuse bone metastases. Three days after receiving denosumab, the patient developed weakness and numbness in his limbs and was diagnosed with drug-induced hypocalcemia due to denosumab. It takes more than 4 months for treating the hypocalcemia in this case with continuous intravenous infusion of calcium gluconate with oral calcium supplementation for 2 months of hospitalization and subsequent 2 months of outpatient treatment with intermittent intravenous infusion of calcium gluconate three times a week along with oral supplementation. Tartrate-resistant acid phosphatase-5b (TRACP-5b), a marker of bone resorption, was a biomarker for the required amount of calcium in this case. Patients with lung cancer with diffuse osteoblastic bone metastases could develop severe hypocalcemia and require long-term calcium supplementation.

## 1 Introduction

Lung cancer is the leading cause of cancer-related death worldwide and is divided into non-small cell lung cancer (NSCLC) and small cell lung cancer (SCLC). Approximately 64 % of NSCLCs are associated with driver gene mutations, including *EGFR* mutations [1]. *EGFR* mutations are the most common actionable driver mutations in patients with NSCLC and occur in 50 % of Asian patients [2]. The standard treatment for EGFR-positive lung cancer is an EGFR tyrosine kinase inhibitor (TKI), with one study showing a progression-free survival (PFS) of 18.9 months and overall survival (OS) of 38.6 months [3].

The frequency of bone metastases in lung cancer is approximately 50 % [4], and bone metastases are treated with zoledronic acid or denosumab to prevent skeletal-related events (SREs), but denosumab often causes hypocalcemia [5]. We describe a patient with severe denosumab-induced hypocalcemia after treatment for bone metastases of lung cancer who required approximately 4 months to recover.

## 2 Case report

**Fig. 1:**
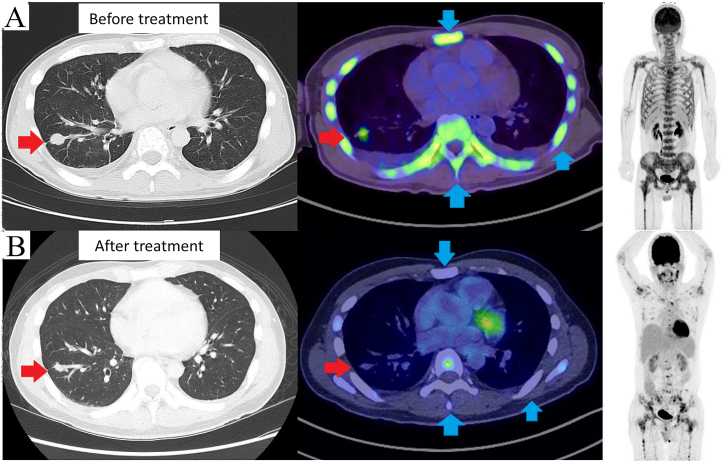
CT and PET/CT scans before treatment (A) show diffusely high FDG uptake, including the mass in the right lower lobe (red arrow), vertebral body, sternum, and ribs (blue arrow). Six months after starting treatment with gefitinib (B), CT and PET/CT scans showed that the mass in the right lower lobe had shrunk, and the diffuse bone metastases had little or no FDG uptake.

**Fig. 2:**
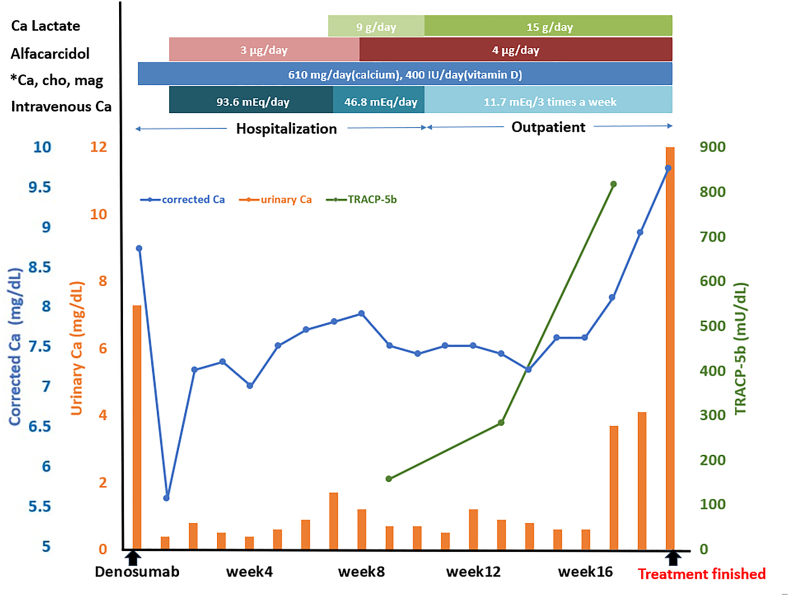
It shows the treatment progress and changes in corrected calcium, urinary calcium, and TRACP-5b levels after denosumab administration. ∗: Calcium carbonate, cholecalciferol, magnesium carbonate.

A non-smoking 48-year-old man presented with clinical stage IVB (cT1bN0M1c) lung adenocarcinoma harboring an *EGFR* L858R mutation, along with diffuse bone metastases in the trunk and proximal limbs and cancerous pleurisy (Fig. 1). The laboratory test results before treatment showed a corrected calcium level of 8.3 mg/dL, urinary calcium level of 7.3 mg/dL, and ALP level of 1353 U/L with normal renal function. The patient was treated with gefitinib 250 mg/d as the first-line treatment. Two days later, denosumab (120 mg) was administered to prevent SREs, with adequate daily supplementation of two combination tablets of precipitated calcium carbonate (containing 610 mg of calcium), cholecalciferol (containing 400 IU of vitamin D), and magnesium carbonate (containing 30 mg of calcium). Three days after the administration of denosumab, tetany and QT prolongation (QTc, 500 ms) were suddenly observed due to denosumab-induced hypocalcemia, which was diagnosed based on laboratory examination (corrected serum calcium level, 5.6 mg/dL and urinary calcium level, 0.4 mg/dL). On d 4, alfacalcidol (3 μg/d) and calcium gluconate hydrate (initial intravenous injection of 7.8 mEq, with subsequent intravenous infusion of 93.6 mEq/d) were started. The corrected serum calcium levels were on an improving trend, but the urinary calcium level did not recover for several weeks. The patient was administered oral calcium lactate (15 g/d) in addition to alfacalcidol (4 μg/d). The intravenous amount of calcium gluconate was gradually reduced and on d 61, after adjusting treatment (11.7 mEq/times) to three times a week, the corrected serum calcium level was maintained. The same treatment was continued at the outpatient clinic after discharge. On d 129 after denosumab administration, the hypocalcemia treatment was completed based on the findings regarding the corrected serum and urinary calcium levels. The patient continued treatment with gefitinib, with a partial response. Regarding bone metabolism markers, the tartrate-resistant acid phosphatase-5b (TRACP-5b), a bone resorption marker, was measured on d 66. When TRACP-5b was measured regularly, it was found to increase gradually. Both the serum and urinary calcium levels were elevated after the increase in TRACP-5b. Interestingly, TRACP-5b had a predictive value for improving calcium levels and supported the drug adjustments for calcium levels (Fig. 2).

## 3 Discussion

We used denosumab to treat diffuse bone metastases from lung cancer and the patient developed severe denosumab-induced hypocalcemia, which required more than 4 months to improve.

Administration of denosumab for diffuse bone metastases from lung cancer rarely leads to severe hypocalcemia. In a previous report, the frequency of hypocalcemia due to denosumab use in cancer with bone metastasis was 5.2 %, with most patients being mild and asymptomatic; however, severe hypocalcemia developed in 2 % [5]. The mechanism by which denosumab leads to hypocalcemia is inhibition of osteoclastic bone resorption and calcium release from the bone, resulting in calcium flow unilaterally into the bone in conditions of high bone turnover [6,7]. When osteoblastic bone metastases are present, denosumab causes severe hypocalcemia. Therefore, severe hypocalcemia requiring prolonged hospitalization has been reported with the use of denosumab in patients with known risk factors such as extensive osteoblastic bone metastasis and a high ALP level [8]. In a previous report on *EGFR* mutation-positive lung cancers with bone metastasis, 84 % of the cases were osteolytic bone metastases, while osteoblastic bone metastases were less common [9]. Our patient was probably at a high risk of denosumab-induced hypocalcemia (at the start of treatment: ALP levels, 1353 U/L). Additionally, previous reports have identified risk factors for severe hypocalcemia, including vitamin D deficiency, renal dysfunction, malignancies such as prostate cancer or SCLC, and the absence of prior use of zoledronic acid [10,11].

In addition, elevated levels of TRACP-5b, a marker of bone resorption, indicate high bone turnover. In a previous study of postmenopausal osteoporosis, there was a rapid and significant decline in TRACP-5b levels after the initiation of denosumab treatment, and the rate of change in TRACP-5b levels correlated with changes in serum calcium levels from baseline to 1 week later, suggesting that it may be useful in predicting the development of hypocalcemia [12]. In this case, the increase in TRACP-5b levels over time indicated that the inhibition of bone resorption was abolished, and the effect of denosumab disappeared. This indicated that calcium could be released from the bones, serum calcium levels and urinary calcium excretion increased, and that TRACP-5b could be correlated with serum calcium levels. Therefore, TRACP-5b levels can be a predictive biomarker for the treatment of hypocalcemia due to denosumab administration.

A previous study reported that the median time to occurrence of grade 2 or higher hypocalcemia following denosumab administration was 3.6 months, while for grade 3 or higher hypocalcemia, it was 4.6 months [10]. Based on our findings and previous studies, serum calcium levels should be monitored 3–7 days after the first dose. If hypocalcemia is present, appropriate management should be tailored to each case. Even if calcium levels stabilize, it is crucial to carefully monitor high-risk patients at monthly intervals during the first 5 months. Long-term follow-up may be necessary for patients with persistent risk factors.

In conclusion, careful monitoring is required in patients with lung cancer with extensive osteoblastic bone metastases, and TRACP-5b levels could be a biomarker for predicting the improvement of hypocalcemia.

## CRediT authorship contribution statement

**Masayuki Mori:** Writing – original draft, Data curation, Conceptualization. **Masayuki Shirasawa:** Writing – review & editing. **Akihito Oguri:** Writing – review & editing. **Hiroki Yamamoto:** Writing – review & editing. **Hideaki Manabe:** Writing – review & editing. **Yoshiro Nakahara:** Writing – review & editing. **Takashi Sato:** Writing – review & editing. **Katsuhiko Naoki:** Writing – review & editing.

## IRB information and informed consent

The retrospective study was approved by the Kitasato University Medical Ethics Organization (B23-156, 23/05/2024), which waived the requirement for patient informed consent.

## Funding

This research did not receive any specific grant from funding agencies in the public, commercial, or not-for-profit sectors.

## Declaration of competing interest

The authors declare that they have no known competing financial interests or personal relationships that could have appeared to influence the work reported in this paper.

## Glossary

NSCLC, non-small cell lung cancer; OS, overall survival; PFS, progression-free survival; SCLC, small cell lung cancer; SREs, skeletal-related events; TKI, tyrosine kinase inhibitor; TRACP-5b, tartrate-resistant acid phosphatase-5b; CT, computed tomography; PET, positron emission tomography; FDG, 2-[(18)F]fluoro-2-deoxy-D-glucose.

## References

[1] Kris M.G., Johnson B.E., Berry L.D. (2014). Using multiplexed assays of oncogenic drivers in lung cancers to select targeted drugs. JAMA.

[2] Paez J.G., Jänne P.A., Lee J.C. (2004). EGFR mutations in lung cancer: correlation with clinical response to gefitinib therapy. Science.

[3] Soria J.C., Ohe Y., Vansteenkiste J. (2018). Osimertinib in untreated EGFR-mutated advanced non-small-cell lung cancer. N. Engl. J. Med..

[4] Katakami N., Kunikane H., Takeda K. (2014). Prospective study on the incidence of bone metastasis (BM) and skeletal-related events (SREs) in patients (pts) with stage IIIB and IV lung cancer-CSP-HOR 13. J. Thorac. Oncol..

[5] Qi W.X., Lin F., He A.N., Tang L.N., Shen Z., Yao Y. (2013). Incidence and risk of denosumab-related hypocalcemia in cancer patients: a systematic review and pooled analysis of randomized controlled studies. Curr. Med. Res. Opin..

[6] Kostenuik P.J., Smith S.Y., Samadfam R., Jolette J., Zhou L., Ominsky M.S. (2015). Effects of denosumab, alendronate, or denosumab following alendronate on bone turnover, calcium homeostasis, bone mass and bone strength in ovariectomized cynomolgus monkeys. J. Bone Miner. Res..

[7] Lau L.H., Cliff E.R.S., Wong V. (2020). Hypocalcaemia following denosumab in prostate cancer: a clinical review. Clin. Endocrinol..

[8] Iizumi S., Shimoi T., Nishikawa T. (2017). Prolonged hypocalcemia following a single dose of denosumab for diffuse bone metastasis of gastric cancer after total gastrectomy. Intern. Med..

[9] Takahara Y., Nakase K., Nojiri M. (2021). Relationship between clinical features and gene mutations in non-small cell lung cancer with osteoblastic bone metastasis. Cancer Treat. Res. Commun..

[10] Body J.-J., Bone H.G., de Boer R.H. (2015). Hypocalcaemia in patients with metastatic bone disease treated with denosumab. Eur. J. Cancer.

[11] Okada N., Kawazoe K., Teraoka K. (2013). Identification of the risk factors associated with hypocalcemia induced by denosumab. Biol. Pharm. Bull..

[12] Asano T., Shimizu T., Takahashi D. (2019). Potential association with early changes in serum calcium level after starting or switching to denosumab combined with eldecalcitol. J. Bone Miner. Metab..

